# Knowledge and risk behaviors related to HIV/AIDS, and their association with information resource among men who have sex with men in Heilongjiang province, China

**DOI:** 10.1186/1471-2458-10-250

**Published:** 2010-05-14

**Authors:** Shengyuan Liu, Kaili Wang, Songpo Yao, Xiaotong Guo, Yancheng Liu, Binyou Wang

**Affiliations:** 1 Department of Epidemiology, Public Health College of Harbin Medical University, Harbin 150081, China; 2 Department of Chronic Disease, Shenzhen Nanshan Center for Chronic Disease Control, Shenzhen 518054, China; 3 Department for viral disease, Heilongjiang Center for Disease Control and Prevention, Harbin 150030, China; 4 Department of Preventive Medicine, Jiamusi University, Jiamusi 154007, China; 5 Department of Thoracic Surgery, the Second Affiliated Hospital of Harbin Medical University, Harbin 150086, China

## Abstract

**Backgroud:**

In Heilongjiang province, the HIV prevalence in men who have sex with men (MSM) is generally lower than other part of China. However, the official perception for their risk of HIV/AIDS infection has been increasing in the province over the years. Moreover, little information on HIV/AIDS was provided to the communities so that we have disadvantage of controlling HIV/AIDS epidemic in the region. The purpose of this study is to investigate the prevalence of HIV among MSM in Heilongjiang province, to assess their knowledge levels and risk behaviors related to HIV/AIDS, and to explore their associations with information resources.

**Methods:**

A cross-sectional study using a standardized questionnaire and blood test was administered in 2008 by local interviewers to a sample (1353) of MSM in four cities in Heilongjiang province.

**Results:**

Among 1353 MSM, 2.3% were identified with HIV infection. About 48.7% of the subjects had multiple male sexual partners and only 37.3% of the subjects had consistent condom use (use every time) in the past 6 months. Most had a fair level of knowledge on HIV/AIDS, with the highest mean knowledge score among the MSM from Jiamusi, those with income 2000-3000 RMB/month, those searching sexual partners via internet and those performed HIV testing over 1 year ago). However, some myths regarding viral transmission (e.g., via mosquito bites or sharing kitchen utensils) also existed. Resources of information from which knowledge and risk behaviors related to HIV/AIDS was most available were television (58.6%) among MSM, followed by sexual partner (51.6%), publicity material (51.0%) and internet (48.7%). Significantly statistical differences of mean knowledge score were revealed in favor of book (*P *= 0.0002), medical staff (*P *= 0.0007), publicity material (*P *= 0.005) and sexual partner (*P *= 0.02). Press (*P *= 0.04) and book (*P *= 0.0003) were contributory to the most frequent condom use (condom use every time), while medical staff (*P *= 0.005) and publicity material (*P *= 0.04) is associated with moderate rate of condom use (condom use often).

**Conclusions:**

Although the prevalence of HIV infection is low among MSM in Heilongjiang province, the situation that the risk behaviors were frequent in the population is alarming. The study suggests that some strategies like condom use and education intervention are practical approaches and need to be strengthened.

## Background

The human immunodeficiency virus (HIV)/acquired immunodeficiency syndrome (AIDS) epidemic has become one of the most serious public health problems globally while Asia has been disproportionately affected by the disease. In China, it was reported that an estimated 740 thousands individuals were infected with HIV and this number is increasing annually[1]. Moreover, it is worse that the original transmission model of HIV/AIDS has changed. In 2009, the Chinese government reported that more than 70% in all new case with HIV/AIDS were infected by sexual transmission that has replaced intravenous drug use as the major route of transmission in China [1]. In sexual transmission, the homosexual transmission accounted for 5.1%, much higher than that of 0.4% in 2005 [2]. Therefore, men who have sex with men (MSM) have become "the potential group" at greater risk of HIV/AIDS transmission because of risk behaviors such as multiple partners and unsafe anal intercourse in China.

To control the situation, public health efforts have been made by strengthening the education programmes to use condom and to advocate reduction of sexual partners number [3], as well as the treatment programmes using antiviral drugs [4]. Although the treatment programmes may prolong the life of patients, the effective education programmes would be more practicable to prevent people from the disease.

However, due to social discrimination and cultural stigma associated with homosexual behaviors in China, the MSM group has to hide their sexual orientation and have little access to the public health care system or educational information [5]. As a result, surveillance among the high-risk MSM population is preliminarily limited. Furthermore, there are limited scientific data about the risky sexual behaviors or educational intervention efforts among MSM in China.

Therefore, the aim of the study was to estimate the prevalence of HIV infection, to evaluate the knowledge about and risk behaviors of HIV/AIDS, as well as their associations with the resource of educational information among MSM in Heilongjiang province, China. The study will provide important guidance for prevention of HIV/AIDS transmission through education, safety measures, voluntary counseling and routine HIV testing.

## Methods

This cross-sectional study was conducted between July and September, 2008 in four cities of Heilongjiang province, China (Harbin, Qiqihar, Mudanjiang and Jiamusi), with approval from the Ethic Committee of Harbin Medical University. The four cities are the top four largest and most populous in Heilongjiang province. There are relatively MSM population in the area thus are optimal site to carry out the study. The study population was the MSM aged at least 15 who had anal intercourse with at least a man in the year before. Because the MSM was a difficult group to contact, samples were collected by population size and location sampling, or "a snowball" sampling. The interviews were conducted in the communities of each city identified with high incidence of homosexuality. Before the interview, a brief description of the study was available to each MSM. If the interviewee agreed to participate, he would answer an anonymous questionnaire face to face with the experienced interviewers for approximately 20 minutes. He would also receive blood testing for HIV antibodies by enzymelinked immunosorbent assay followed by confirmatory Western blot.

Concerned questionnaires content was detailed as the following: 1. sociodemographic characteristics (like age, marital status, race, education, employment and economic status), circumstance of knowing the sex partners and HIV testing status; 2. general awareness of HIV/AIDS: (Q1) May a person who looks healthy carry HIV? (Q2) Can a person be infected with HIV by transmitting blood or blood products? (Q3) Can a person be infected by HIV when sharing needles with HIV carriers? (Q4) Can condom use reduce the risk of HIV spread? (Q5) Can the risk of HIV spread be reduced by maintaining one sexual partner with uninfected HIV? (Q6) May pregnant women with HIV transmit the virus to their children? (Q7) Can HIV be transmitted by eating together with HIV carriers or patients with AIDS? (Q8) Can HIV be transmitted by Mosquito bites? These questions were correctly answered as "yes", but wrongly answered or unknown as "no"; 3. risk behaviors (number of partners, type of partner, and frequency of condom use); and 4. information resources (television, radio, press, book, medical staff, publicity material, internet, school and sexual partner).

The MSM's knowledge of AIDS was assessed using a mean score developed from eight questions regarding HIV transmission and concepts. One point was given for each correct answer and 0 points for each incorrect or the unknown answer.

The MSM were informed that their participation of the questionnaire was entirely voluntarily so that they were fine not to answer the questions too private to them. The information obtained would be saved confidentially with no names or identifying information appear in publications. An informed consent was obtained from each participant.

The primary analysis investigated the knowledge, risk behaviors and information resources. For the comparative aspect of the objectives, ANCOVA analyses were performed for quantitative variables. The logistic regression analyses were performed for qualitative variables, which had controlled confounding factors. Participants with missing data on any of the critical information were excluded from these analyses. All data analyses were performed by using SPSS 13.0 and a *P *value of less than 0.05 was considered to be statistically significant.

## Results

### Demographic characteristics and information resources

As shown in the Table 1, of the MSM population, 451 were from Harbin, 300 were from Qiqihar, 300 were from Mudanjiang and 302 were from Jiamusi. About 44.4% of the MSM were age 25 or below and about 97.4% of the MSM had reached middle school education or above. Clearly, most MSM (71.2%) chose 'single' as their marital status as a major option. The majorities of the participants were in the form of blue collar (52%) and were below 1000 RMB per month (54.5%). Data analyzed also indicates that the largest number (41%) searched for sexual partners from internet. There is a similar proportion between the tested (41%) and the never tested (59%) over 1 year ago. The main resources of information accessible to the MSM were television (58.6%), sexual partner (51.6%), publicity material (51.0%) and internet (48.7%). Most MSM exposed to HIV/AIDS information had received this information from multiple resources.

**Table 1:** Mean Knowledge Score by social-demographic characteristics of MSM.

	N(%)	Means	Std	P value
City				<0.0001
Harbin	451(33.3)	6.80	1.53	
Qiqihar	300(22.2)	7.16	1.32	
Mudanjiang	300(22.2)	6.78	1.65	
Jiamusi	302(22.3)	7.50	0.81	
Age group				0.0001
15-25 years	601(44.4)	7.03	1.39	
26-35 years	357(26.4)	6.98	1.44	
36-45 years	281(20.8)	7.28	1.18	
46+ years	114(8.4)	6.59	1.82	
Marriage status				0.87
Single	963(71.2)	7.02	1.41	
Married and Cohabitation	294(21.7)	7.04	1.45	
Divorce and Widowed	96(7.1)	7.10	1.33	
Education				<0.0001
≤1 education	35(2.6)	5.51	2.44	
2 education	325(24)	6.73	1.70	
3-4 education	582(43)	7.05	1.29	
≥5 education	411(30.4)	7.37	1.03	
Vocation				0.17
Unemployed	219(16)	6.91	1.45	
Students	295(22)	7.14	1.28	
White collar	137(10)	7.17	1.30	
Blue collar	702(52)	7.00	1.48	
Income (RMB per month)				0.005
<1000	738(54.5)	6.92	1.50	
1000-2000	447(33)	7.17	1.31	
2000-3000	132(9.8)	7.24	1.17	
>3000	36(2.7)	6.81	1.53	
Places of searching sex partners				<0.0001
Bar/nightclub	329(24)	7.21	1.17	
Bath/Saunas	280(21)	6.68	1.65	
Park/latrine	146(11)	6.67	1.75	
Internet	560(41)	7.22	1.22	
Fixed locations	38(3)	6.76	1.92	
HIV testing over 1 year ago				<0.0001
Tested	558(41)	7.39	1.08	
Never tested	795(59)	6.78	1.56	

### Knowledge on HIV/AIDS

On awareness of three transmission routes of HIV, many subjects gave several answers. The "blood transmission (Q2 and 3)" was chosen the most by 95.3%; the second most was "mother to child (Q6)" with 93.1%; and 89.5% chose "sexual transmission (Q4 and 5)". When asked whether HIV can be transmitted by eating together with HIV carriers or patients with AIDS, more than three fourths of the sample selected the false transmission route. Regarding other knowledge of HIV, participants were significantly more likely to endorse (71.5%) than refute (28.5%) the misconception that HIV could be transmitted via a mosquito bite. Table 1 summarizes the mean knowledge score about HIV/AIDS in MSM population and *P *values. Classification by city, age, education, income, places of searching sexual partners, HIV testing showed a statistically significance for mean knowledge score (the MSM from Jiamusi, those whose income was 2000-3000, those searching sexual partners from internet and those performed HIV testing over 1 year ago had a good mean knowledge score; however, the MSM who were more than 46 years old and those with less than 1 education had a poor mean knowledge score about HIV/AIDS).

### Risk behaviors related to HIV/AIDS

Among those (1134) who had anal intercourse with male sexual partners in the past 6 months, 41.9% had a male sexual partner and 58.1% had more than one male sexual partner. The MSM population from Qiqihar, those searching sexual partners from fixed location and those performed HIV testing over 1 year reported significantly more anal intercourse with a male sexual partner in the past 6 months and the MSM from Harbin, those aged from 25 to 34 years and those searching sexual partners from Bath/Saunas in Harbin reported significantly more anal intercourse with multiple male sexual partner in the past 6 months. It can be seen that the frequency of condom use was highly dependent on the places of searching sexual partners where the MSM searching sexual partners from Bar/nightclub (50.38%) had the most frequent use (*P*_*unadjusted *_= 0.0006). The group of 25 to 34 years gave a more attention to condom use every time, but the group older than 45 years gave the least attention to the safety intervention (*P*_*unadjusted *_= 0.01). Most single MSM (46.76%) kept a safe behavior (consistent condom use) each time having an anal intercourse with partners every time (*P*_*unadjusted *_= 0.04). Although the MSM from different vocations showed no significantly statistical difference in consistent condom use (use condoms every time), those with income between 2000 and 3000 RMB/month had the safer sexual behaviors due to consistent condom use (*P*_*unadjusted *_= 0.01). The MSM who did HIV test had higher frequency of condom use than those who were never tested over 1 year (*P*_*unadjusted *_< 0.0001). Multivariate logistic regression adjusted for city, age, education, marriage status, vocation, income, places of searching sexual partners and HIV testing history was used to assess these associations. The MSM (those in Qiqihar [*P*_*adjusted *_< 0.0001] and those searching sexual partners from fixed locations [*P*_*adjusted *_= 0.0003]) had more anal intercourse with a male sexual partner in the past 6 months. The MSM (those in Harbin [*P*_*adjusted *_< 0.0001], those aged 25-34 years [*P*_*adjusted *_= 0.003] and those searching sexual partners from bath/saunas [*P*_*adjusted *_< 0.0001]) had more anal intercourse with more than one male sexual partner in the past 6 months (Table 2). The MSM (those whose income was 2000-3000 RMB/month [*P*_*adjusted *_= 0.02], those searching sexual partners from bar/nightclub [*P*_*adjusted *_= 0.02], and those tested HIV status [*P*_*adjusted *_< 0.0001]) had more consistent condom use (Table 2).

**Table 2:** Results of multivariate and multiple logistic regression analysis of factors related to risk behaviors among MSM population.

	Ever have had anal intercourse behavior in the past 6 months		Had anal intercourse with a male sex partner in the past 6 months		Had anal intercourse with multiple male sex partner s in the past 6 months		Used a condom every time	
	
	**OR **_**adjusted**_ (95% CI)	*P*	**OR **_**adjusted**_ (95% CI)	*P*	**OR **_**adjusted**_ (95% CI)	*P*	**OR **_**adjusted**_ (95% CI)	*P*
City		<0.0001		<0.0001		<0.0001		NS
Harbin	1		1		1		1	
Qiqihar	1.79 (1.11-2.90)		3.44 (2.46-4.81)		0.43 (0.31-0.59)		0.80 (0.57-1.13)	
Mudanjiang	0.51 (0.34-0.77)		1.87 (1.33-2.64)		0.39 (0.29-0.54)		0.78 (0.55-1.11)	
Jiamusi	1.06 (0.67-1.68)		2.43 (1.70-3.46)		0.48 (0.34-0.67)		0.67 (0.47-0.96)	
Age group		0.006		NS		0.003		NS
15-24 years	1		1		1		1	
25-34 years	1.29 (0.82-2.05)		0.79 (0.57-1.10)		1.38 (1.01-1.90)		1.11 (0.79-1.56)	
35-44 years	0.61 (0.35-1.08)		0.74 (0.47-1.16)		1.00 (0.65-1.53)		0.83 (0.52-1.34)	
45+ years	0.43 (0.21-0.86)		1.02 (0.57-1.83)		0.56 (0.32-0.98)		0.63 (0.33-1.19)	
Education		NS		NS		NS		NS
≤1 education	1		1		1		1	
2 education	1.04 (0.43-2.49)		1.05 (0.47-2.32)		1.01 (0.49-2.11)		2.15 (0.86-5.38)	
3-4 education	1.54 (0.65-3.67)		1.35 (0.61-2.96)		1.02 (0.50-2.09)		1.84 (0.74-4.53)	
≥5 education	1.37 (0.56-3.40)		1.23 (0.55-2.75)		1.04 (0.50-2.19)		2.06 (0.82-5.19)	
Marriage status		NS		NS		NS		NS
Single	1		1		1		1	
Married and cohabitation	0.93 (0.56-1.52)		1.12 (0.75-1.66)		0.87 (0.60-1.27)		0.88 (0.58-1.34)	
Divorce and widowed	1.70 (0.82-3.50)		1.43 (0.83-2.45)		0.99 (0.59-1.66)		0.85 (0.48-1.51)	
Vocation		NS		NS		NS		NS
Unemployed	1		1		1		1	
Students	0.94 (0.54-1.66)		1.11 (0.73-1.69)		0.89 (0.59-1.33)		1.06 (0.68-1.64)	
White collar	0.40 (0.21-0.79)		1.20 (0.71-2.03)		0.51 (0.31-0.85)		1.05 (0.60-1.82)	
Blue collar	0.66 (0.40-1.09)		1.13 (0.77-1.66)		0.71 (0.49-1.02)		1.25 (0.84-1.86)	
Income(RMB/month)		0.02		NS		NS		0.02
<1000	1		1		1		1	
1000-2000	1.35 (0.90-2.02)		0.86 (0.62-1.20)		1.35 (0.98-1.85)		1.03 (0.73-1.46)	
2000-3000	2.79 (1.39-5.58)		0.94 (0.58-1.52)		1.74 (1.10-2.75)		1.93 (1.19-3.14)	
>3000	3.27 (0.92-11.58)		1.00 (0.45-2.23)		1.79 (0.84-3.82)		0.74 (0.33-1.62)	
Places of searching sexual partners		0.04		0.0003		<0.0001		0.02
Bar/nightclub	1		1		1		1	
Bath/Saunas	1.91 (1.20-3.03)		0.72 (0.49-1.05)		1.92 (1.34-2.74)		0.69 (0.47-1.02)	
Park/latrine	1.20 (0.69-2.06)		0.98 (0.62-1.55)		1.11 (0.72-1.71)		0.43 (0.26-0.71)	
Internet	1.64 (1.10-2.43)		1.36 (1.00-1.84)		0.99 (0.74-1.33)		0.81 (0.58-1.12)	
Fixed locations	1.60 (0.53-4.83)		3.02 (1.47-6.21)		0.44 (0.21-0.92)		0.57 (0.27-1.22)	
HIV testing over 1 year ago		0.01		NS		NS		<0.0001
Tested	1		1		1		1	
Never tested	0.66 (0.48-0.92)		0.82 (0.64-1.04)		0.97 (0.77-1.23)		0.59 (0.46-0.76)	

Note: NS = not statistical significance

### Relationship of knowledge and risk behaviors of HIV/AIDS to resources of information

Additional file 1 showed the association of HIV/AIDS variables with various resources of information. Because the socio-demographic characteristics of the subjects may affect the association between the variables and the information resources, the analysis was done with adjustments on city, age groups, marital status, education, vocation, income, places of searching sexual partners and HIV testing.

Knowledge obtained from book (*P *= 0.0002), medical staff (*P *= 0.001), publicity material (*P *= 0.01) and sexual partner (*P *= 0.02) was independently associated with possessing a better mean knowledge score related to HIV/AIDS. Exposure to radio (*P *= 0.001) and sexual partner (*P *= 0.03) was respectively related to more frequent sexual behaviors.

Behavioral change on condoms use during anal intercourse with male sexual partners has been investigated by analyzing the association between resources of information and the frequency of condom use in the last 6 months. Press (*P *= 0.04) and book (*P *= 0.0003) were found to be associated with a safer sexual behavior that is defined as condom use every time, Medical staff (*P *= 0.005) and publicity material (*P *= 0.04) were associated with moderate rate of condom use (condom use often).

### HIV infection

In the study, 31 (2.3%) subjects were tested positive for HIV with the proportion of 64.5% from Harbin, 22.6% from Qiqihar, 3.2% from Jiamusi and 9.7% from Mudanjiang. Among these 31 MSM, 8 were aware of their positive status but the rest did not know until tested in the study. Six with known history of HIV used condoms when having sex in the past 6 months. Among the 23 who did not know about their HIV status before the study, two had unprotected sex with no condom use every time.

## Discussion

This study is unique in that it provides the first empirical data available on knowledge and risk behaviors of HIV/AIDS, as well as their associations with information resources among men who have sex with men (MSM) in Heilongjiang province, China

This study used a sample of 1353 men who have sex with men aged 19-60 years to estimate HIV prevalence, to evaluate their knowledge and risk behaviors of HIV/AIDS, and to explore their associations with information resources. Regarding basic knowledge of HIV/AIDS, more than two thirds of the MSM maintained accurate knowledge on some aspect of HIV transmission, but some false beliefs that HIV/AIDS can be transmitted by mosquito bites or dining together still existed. Generally, compared to other studies [6-10] in China, the MSM in the study had a fair level of knowledge. Among different recruitment cities, the Jiamusi city had the highest knowledge level in the four cities, which may be accounted for by timely information and effective program. In the study, the oldest age group (≥46 years) had a poor mean knowledge score, which may be due to the fact that at the time this group did not have enough exposure on HIV/AIDS in school,. Also since the emergence of HIV/AIDS, these people have not been adequately educated on the disease. The MSM in higher education had a better mean knowledge score, which may be explained by the fact that the MSM in higher education level may have more chances to obtain the information pertaining to the disease than those in the lower education level. Although the MSM from different vocations had no statistically significant difference in mean knowledge score, the middle-income population had a good mean score, which may be due to their particularly large number and easy access to more information. The internet provided such important approach to communicate [11,12] that many MSM may acquire knowledge on HIV/AIDS when searching sexual partners via it, which resulted in a high mean score in this population. The people tested for HIV over 1 year ago had significantly higher mean knowledge score than those who were never tested. This may be accounted for the fact that when these people were tested for HIV antibody, they may have a lesson relevant to CDC workers-led HIV education.

Knowledge and experience about risk behaviors appeared mixed. The majority of MSM population knew that condom use was effective against the transmission of HIV and that intercourse with a fixed sexual partner with uninfected HIV can reduce the risk of HIV spread. However, majority of MSM population did not use condom consistently when they had anal intercourse with multiple sexual partners.

The study revealed that there was a statistically significant difference in exposure to sexual behaviors among these cities. This holds true among MSM population in Heilongjiang province, suggesting that the observed geographical differences in sexual behavior may be caused by the different characteristics of the MSM in different cities. Therefore, specific interventions designed to target the social and cultural milieu of different geographical locations where sex acts take place appear essential and it is unwise to promote the same safer sex measures among different societal contexts. The prevalence of consistent condom use in the four cities was so low that it is necessary to increase the strength of education on condom use and condom release.

The evidence showed that the MSM of 15 to 44 years were at high risk for sexual behavior due to anal intercourse with multiple sexual partners in the past 6 months. This may be explained by the fact that the MSM were sexually active in the so called young people (less than 44 years was the young people, as provided by the United Nations standard), which intend to have frequent sex intercourse than other age groups. Therefore, it is necessary to actively guide their sexual behaviors and correct the homosexuality in time when they were younger and the sexual perversion was mild.

Approximately one third of MSM in the study reported to live with a woman or have been married to a woman. The high rates of marriage to a woman or having both female and male sexual partners concurrently among Chinese MSM might be due to the pressure from society and family [5]. To avoid the pressure, MSM might engage in heterosexual behaviors to keep the secret homosexual preferences and practices, which may increases the spreading of HIV/AIDS to their female partners and further to the general Chinese population (as shown in our results, statistical difference is not found among marriage status, which further demonstrated that marriage was an umbrella that the homosexual preferences and practices hide under). Therefore, HIV prevention efforts targeting MSM should focus on correcting risky behaviors, and on improving the skills dealing with the social pressures and stigmatizing perceptions.

In the study, there was statistical significance for risk behaviors among different places of searching sexual partners, Bath/Saunas of which can be likened to the concentric circle where the risk behavior with more than one sexual partner was frequent among MSM and park/latrine can be the gathering place where consistent condom use was scarce when having risky sexual behaviors. The phenomena can be explained as Bath/Saunas were active sex places, where commercial and noncommercial sexual behaviors often occurred, resulting in frequent sexual behaviors with more partners. However park/latrine were hidden places where casual sexual behavior occurred with no condoms, which led to inconsistent condom use. Thus, it is necessary to strengthen the management of these places and to increase the condom release location.

The Chinese government has offered free HIV antibody testing, which has achieved good effects to control HIV epidemic [13]. However, in our study, it was men with HIV testing over 1 year who reported more risky sexual behaviors and more likelihood of unsafe anal intercourse with partners among MSM in the past 6 months, which demonstrated that knowledge of negative HIV serostatus may make MSM have unbridled sexual behaviors. Therefore, when testing HIV antibody, MSM should concomitantly be educated on HIV risks.

Fortunately, despite the high levels of risky behavior, HIV among MSM remains relatively low in both prevalence (2.3%) and absolute numbers which presents a window of opportunity for prevention. However, compared to the data in other years in Heilongjiang province [14,15], the HIV prevalence has actually increased in MSM population. The increasing prevalence of HIV among MSM may predict a more general increase of HIV in the population over the next several years. Although the current rate of HIV infection in MSM is in the acceptable scope, the increasing rate of HIV in MSM and the increasing number of MSM suggest a possible route for the virus to spread across these vulnerable groups. Given the potential high risk of MSM contracting HIV, it is alarming that the increased HIV rates may predict future spread of HIV in the general population. Therefore, more interventions should be warranted to minimize risk for HIV in the MSM population.

The study indicated that messages related to HIV/AIDS have reached the MSM population by various information resources. Of individual resources, the television was frequently cited by the majority of MSM, followed by information from sexual partner, publicity material and internet in the study, which was different from results in previous studies [16].

Our results also suggested that increasing interpersonal contact might be of great advantage for MSM to gain more HIV/AIDS knowledge and to reduce risky sexual behaviors in Heilongjiang province. For instance, contact with medical staffs and sexual partners appears very effective in the education of the MSM (the two information resources showed a higher knowledge score) and communication with medical staffs implicates a safer sexual behaviors (condom use every time or often). This may be explained that interpersonal communication, guaranteeing deeper understanding and sharing of personal experiences, has great advantages in transmitting information. Exposure to information on HIV/AIDS through book was related to a higher mean knowledge score and more frequent condom use. This may be related to the more detailed information of HIV/AIDS described in books that made the MSM easy to understand. Our results, consistent with changes demonstrated elsewhere [17], suggest the positive impact of the role of community intervention programmes (e.g. brochures, booklets and pamphlets were all called publicity material) on HIV/AIDS-related knowledge and behaviors. Of these programmes, publicity material plays a very important role partly because of its low price, its clear intent and its convenience.

Some limitations should be noted in considering the findings of this study:

1. Selective bias may have limited generalizability to the entire MSM populations of these regions;

2. Some cultural factors such as shame and stigma on admitting homosexuality may have affect the recruitment of study subjects to disclose their sexual behaviors so that nonparticipation and nonresponse may have influenced the validity of the study;

3. Information bias may limit subjects to provide accurate information on certain sensitive issues such as sexual behaviors;

4. Some questions may result in recall bias;

5. This was a cross-sectional study that may prevent us from defining the causal relationship between predictors and HIV/AIDS-related knowledge and risk behaviors.

Despite these limitations, the study has several strengths:

1. It identified that a younger (25-34 years) MSM population, a MSM population searching sexual partners from Bath/Saunas and a MSM population lived in Harbin are all more likely to have multiple sexual partners;

2. It revealed that a MSM population with prior HIV testing, a middle-income MSM population and a MSM population searching sexual partners from Bar/nightclub are all more likely to engage in sexual risk behaviors (inconsistent condom use);

3. It also showed that a few resources of information of HIV/AIDS are more effective to the MSM population. Of note, the availability and use of information resources about HIV/AIDS are not sufficient enough to assess the understanding about HIV/AIDS among MSM population. Each of the information resources is limited to account for only certain degree of awareness about the pandemic.

## Conclusion

Given the results presented in this study, a reinvigorated and tailored approach to HIV prevention among MSM in China is urgently needed. Prevention efforts should be focused on improving social awareness on HIV/AIDS, on enforcing education strategy to reduce sexual risk and on improving the strength and quality of counseling. This study provided will enable public health experts to develop specific HIV/AIDS intervention programs for the high risk population (such as MSM) in China.

## Competing interests

The authors declare that they have no competing interests.

## Authors' contributions

LSY drafted the manuscript, participated in the collection of data, participated in the design of the study and performed the statistical analysis. WKL participated in the collection of data. YSP participated in the collection of data, participated in the design of the study and performed the statistical analysis. GXT participated in the draft of the manuscript. LYC participated in the collection of data. WBY designed the study, and participated in coordination and helped to draft the manuscript. LSY, WKL, YSP and GXT have made equally significant contributions to this manuscript.

## Pre-publication history

The pre-publication history for this paper can be accessed here:

http://www.biomedcentral.com/1471-2458/10/250/prepub

## Supplementary Material

Additional file 1 **Difference of HIV/AIDS-related variables according to sources of information**. The file contains the data about HIV/AIDS-related variables and sources of information. Click here for file
