# Supplementary material for: Knowledge and risk behaviors related to HIV/AIDS, and their association with information resource among men who have sex with men in Heilongjiang province, China
Source: BMC Public Health. 2010 May 14;10:250. doi: 10.1186/1471-2458-10-250 (PMC2885333; doi:10.1186/1471-2458-10-250)
Supplement: Additional file 1 — Difference of HIV/AIDS-related variables according to sources of information. The file contains the data about HIV/AIDS-related variables and sources of information. [file 1471-2458-10-250-S1.DOC]

Additional table: Difference of HIV/AIDS-related variables according to sources of information

|  | Source of information | | | | | | | | | | | | | | | | | |
| --- | --- | --- | --- | --- | --- | --- | --- | --- | --- | --- | --- | --- | --- | --- | --- | --- | --- | --- |
|  | Television | | Radio | | Press | | Book | | Medical staff | | Publicity material | | Internet | | School | | Sexual partner | |
|  | Yes | No | Yes | No | Yes | No | Yes | No | Yes | No | Yes | No | Yes | No | Yes | No | Yes | No |
| Knowledge of HIV/AIDS score |  |  |  |  |  |  |  |  |  |  |  |  |  |  |  |  |  |  |
| Number | 793 | 560 | 433 | 920 | 405 | 948 | 406 | 947 | 265 | 1088 | 690 | 663 | 659 | 694 | 98 | 1255 | 698 | 655 |
| Means of scores | 7.09 | 6.94 | 7.09 | 7.01 | 7.16 | 6.98 | 7.28 | 6.93 | 7.45 | 6.93 | 7.22 | 6.83 | 7.15 | 6.92 | 7.23 | 7.02 | 7.12 | 6.94 |
| Standard deviation | 1.37 | 1.47 | 1.32 | 1.46 | 1.18 | 1.50 | 1.08 | 1.52 | 0.92 | 1.49 | 1.25 | 1.54 | 1.30 | 1.50 | 1.26 | 1.42 | 1.32 | 1.50 |
| P value | NS |  | NS |  | NS |  | 0.0002 |  | 0.001 |  | 0.005 |  | NS |  | NS |  | 0.02 |  |
| Ever have had anal intercourse  behavior in the past 6 months |  |  |  |  |  |  |  |  |  |  |  |  |  |  |  |  |  |  |
| Yes | 652 | 482 | 337 | 797 | 333 | 801 | 345 | 789 | 233 | 901 | 586 | 548 | 564 | 570 | 86 | 1048 | 604 | 530 |
| No | 141 | 78 | 96 | 123 | 72 | 147 | 61 | 158 | 32 | 187 | 104 | 115 | 95 | 124 | 12 | 207 | 94 | 125 |
| P value | NS |  | 0.001 |  | NS |  | NS |  | NS |  | NS |  | NS |  | NS |  | 0.03 |  |
| Had anal intercourse with a male  sex partner in the past 6 months |  |  |  |  |  |  |  |  |  |  |  |  |  |  |  |  |  |  |
| Yes | 291 | 184 | 146 | 329 | 141 | 334 | 144 | 331 | 110 | 365 | 252 | 223 | 237 | 238 | 38 | 437 | 249 | 226 |
| No | 502 | 376 | 287 | 591 | 264 | 614 | 262 | 616 | 155 | 723 | 438 | 440 | 422 | 456 | 60 | 818 | 449 | 429 |
| P value | NS |  | NS |  | NS |  | NS |  | NS |  | NS |  | NS |  | NS |  | NS |  |
| Had anal intercourse with multiple male sex partners in the past 6 months |  |  |  |  |  |  |  |  |  |  |  |  |  |  |  |  |  |  |
| Yes | 361 | 298 | 191 | 468 | 192 | 467 | 201 | 458 | 123 | 536 | 334 | 325 | 327 | 332 | 48 | 611 | 355 | 304 |
| No | 432 | 262 | 242 | 452 | 213 | 481 | 205 | 489 | 142 | 552 | 356 | 338 | 332 | 362 | 50 | 644 | 343 | 351 |
| P value | NS |  | NS |  | NS |  | NS |  | NS |  | NS |  | NS |  | NS |  | NS |  |
| Condom use when anal intercourse  in the past 6 months |  |  |  |  |  |  |  |  |  |  |  |  |  |  |  |  |  |  |
| No use | 47 | 51 | 20 | 78 | 19 | 79 | 13 | 85 | 9 | 89 | 38 | 60 | 36 | 62 | 4 | 94 | 48 | 50 |
| Often use | 322 | 209 | 177 | 354 | 152 | 379 | 154 | 377 | 123 | 408 | 290 | 241 | 265 | 266 | 38 | 493 | 294 | 237 |
| Every time | 283 | 221 | 140 | 364 | 162 | 342 | 178 | 326 | 101 | 403 | 257 | 247 | 262 | 242 | 44 | 460 | 261 | 243 |
| P value | NS |  | NS |  | 0.04 |  | 0.0003 |  | 0.005 |  | 0.04 |  | NS |  | NS |  | NS |  |

Note: NS=not statistical significance
